# Research on the mechanism of *Bacillus velezensis* A-27 in enhancing the resistance of red kidney beans to soybean cyst nematode based on TMT proteomics analysis

**DOI:** 10.3389/fpls.2024.1458330

**Published:** 2024-09-23

**Authors:** Yi Hu, Yibing Ma, Liyi Wang, Qingqing Luo, Zengqi Zhao, Jianming Wang, Yumei Xu

**Affiliations:** ^1^ Laboratory of Nematology, Department of Plant Pathology, College of Plant Protection, Shanxi Agricultural University, Jinzhong, China; ^2^ Inveterate Group, Systematics, Manaaki Whenua-Landcare Research, Auckland, New Zealand; ^3^ Shanxi Key Laboratory of Integrated Pest Management in Agriculture, Shanxi Agricultural University, Taiyuan, China

**Keywords:** red kidney bean, SCN, Bacillus velezensis A-27, JA biosynthesis, TMT, induced systemic resistance (ISR)

## Abstract

Soybean cyst nematode (SCN) poses a significant challenge to red kidney beans cultivation, resulting in yield losses and quality deterioration. This study investigates the molecular mechanisms using Tandem Mass Tag (TMT) based proteomics technology to explore how the plant growth-promoting rhizobacterium (PGPR) *Bacillus velezensis* A-27 enhances the resistance of red kidney beans against SCN. The results revealed that out of 1,374 differentially expressed proteins (DEPs) in the red kidney beans roots, 734 DEPs were upregulated and 640 DEPs were downregulated in the A-27 + J2 *vs* J2 treatment group. KEGG analysis revealed that 14 DEPs were involved in the α-LeA metabolic pathway, crucial for the biosynthesis of jasmonic acid (JA) in plants. Quantitative real-time PCR (qRT-PCR) confirmed the upregulation of 4 key genes (*PLA1, AOS*, *AOC*, *ACX*) in the JA biosynthesis pathway, while enzyme-linked immunosorbent assay (ELISA) demonstrated a significant increase in JA content in the roots. The study demonstrates that *B. velezensis* A-27 stimulates induced systemic resistance (ISR) in red kidney beans, and induce JA biosynthesis by regulating the expression of key enzymes in the α-LeA metabolic pathway. This enhances the plant’s defense against SCN, providing a theoretical foundation for the potential use of *B. velezensis* A-27 as a biocontrol agent for managing SCN in leguminous crops.

## 1 Introduction

Red kidney bean (*Phaseolus vulgaris* L.) is a cultivated variety of common bean, from the legume family, with a dwarf erect plant. Introduced to Lan county, Shanxi Province from Italy in 1992, it has become a extensively cultivated cereal crop in the region (Han et al., 2016). Originally from Mexico and Argentina, this crop is highly popular among consumers locally and globally due to its economic value. By 2018, the planting area in Lan county alone had reached 8,667 hm^2^, ranking first nationwide in cultivation and export scale. Apart from Shanxi Province, red kidney beans are also grown in Heilongjiang, Shaanxi, Yunnan, and other regions in China (Hao et al., 2019).

In recent years, the extensive cultivation and field management practices have led to a significant issue with soybean cyst nematode (SCN), which has become a major limiting factor affecting the yield and quality of red kidney beans. SCN infestation can cause yield reductions of 20% to 30% in mild cases and complete crop failure in severe instances. Additionally, the feeding site created by SCN can pave the way for root rot pathogens to invade, resulting in compound infections that severely hinder the development scale and export quality of the red kidney bean industry (Duan et al., 2011).

Plant growth-promoting rhizobacteria (PGPR), such as *Bacillus firmus* (Terefe et al., 2009), have demonstrated potential as biocontrol agents against plant-pathogenic nematodes (PPNs) (TariqJaveed et al., 2021). Studies have shown that *B. firmus* (Seo and Lee, 2004), *Klebsiella pneumoniae* (Kang et al., 2018; Liu et al., 2018), *B. amyloliquefaciens* (Jamal et al., 2017), *B. subtilis* (Cao et al., 2019), *B. atrophaeus* (Ayaz et al., 2021), *B. cereus* (Yin et al., 2021), and *B. altitudinis* (Jiao et al., 2022) can stimulate plants to develop induced systemic resistance (ISR), thereby enhancing their defense against PPNs. *Bacillus velezensis*, as PGPR, dedicates over 9% of its genome to the synthesis of various metabolic intermediates (Adeniji et al., 2019), which include antibiotics, antioxidants, antifungal factors, and growth promoters. The antimicrobial compounds produced by *B. velezensis* consist mainly of bacteriocins, polyketides, and lipopeptides (Jamal et al., 2017; Rabbee et al., 2019; Heo et al., 2022). These compounds not only inhibit the growth of certain bacteria by disrupting their cell walls or membranes but also suppress the growth of pathogenic fungal hyphae and spore germination (Rabbee and Baek, 2020). Flagellin or secondary metabolites from *B. velezensis* can trigger ISR in plants (Tian et al., 2022), enhancing their defensive capabilities against pathogenic microbes and nematodes, thus reducing plant diseases. These compounds can bind to receptor proteins on the cell membrane, initiating primary signals that activate systemic signal transduction pathways (JA, ethylene and SA pathway). This stimulation leads to the production of defense responses in plants such as the secretion of antimicrobial enzymes, reinforcement of cell walls, secretion of plant defense compounds, and activation of the lipoxygenase pathway. These responses help plants in combating pathogen infection (Ayaz et al., 2021; She et al., 2021).

Jasmonates (JAs) are lipid-derived stress hormones synthesized by plants, playing a crucial role in regulating plant responses to biotic stresses, including defense against pathogen infection and herbivore damage (Wasternack and Hause, 2013; Kammerhofer et al., 2015; Wasternack and Strnad, 2016). The biosynthesis of JAs begins with alpha-linolenic acid (α-LeA) through the octadecanoid pathway (Kombrink, 2012). In plastids, α-LeA is initially produced through the coordinated actions of fatty acid desaturase (FAD) and phospholipase A1 (PLA). It is then converted to 13-hydroxy-9,11,15-trienoic acid (13-HPOT) by 13-lipoxygenase (LOX), followed by transformation into 12,13-epoxy-9,11,15-trienoic acid (12, 13-EOT) through the activities of allene oxide synthase (AOS) and hydroperoxide dehydratase (HPL). Subsequently, it is converted to 12-oxo-phytodienoic acid (OPDA) by allene oxide cyclase (AOC). OPDA is then transported to peroxisomes, where it is reduced to 3-oxo-2-(cis-2’-pentenyl)-cyclopentane-1-octanoic acid (OPC-8) by OPDA reductase (OPR). OPC-8 is further activated to OPC-8 CoA by OPC-8 CoA ligase (OPCL) and subsequently transformed into JA through three rounds of β-oxidation catalyzed by various enzymes, including acyl-CoA oxidase (ACX), multifunctional protein (MFP), and 3-ketoacyl-CoA thiolase (KAT) (Wasternack and Hause, 2013; Wasternack and Strnad, 2016).

JA, as a principal plant defense hormone, plays a crucial role in plant resistance against PPNs (Liu et al., 2022). Research has shown that JA signaling is involved in defense mechanisms against root-knot nematodes such as *Meloidogyne incognita* (Huang et al., 2022), *M. chitwoodi* (Vieira dos Santos et al., 2013), and *M. graminicola* (Nahar et al., 2011), as well as the cyst nematode *Heterodera schachtii* (Kammerhofer et al., 2015). Treatment with methyl jasmonate (MeJA) has been found to induce resistance to *M. graminicola* in rice (Nahar et al., 2011; Kumari et al., 2015). The accumulation of JA precursor cis-c-12-oxo-phytodienoic acid (OPDA), along with JA or jasmonoyl isoleucine (JA-IIe), enhances *Arabidopsis* resistance against *M. hapla* (Gleason et al., 2016). Moreover, overexpressing *Arabidopsis* JA biosynthesis genes in soybean plants offers some defense against SCN (Olson-Manning et al., 2015). Conversely, SCN infection can suppress both JA biosynthesis genes and JA signaling response in soybean plants (Ithal et al., 2007).

To investigate the molecular mechanism by which *B. velezensis* enhances the resistance of red kidney beans to SCN disease. Tandem Mass Tag (TMT) proteomic technology was employed to compare and analyze the differentially expressed proteins (DEPs) under different treatments, followed by corresponding bioinformatics analysis. Quantitative real-time PCR (qRT-PCR) was used to analyze the changes in the transcriptional levels of key genes in the JA synthesis pathway, and the content of JA in plant roots was quantified. These experiments and analyses provide theoretical support for the management of SCN through the use of *B. velezensis*.

## 2 Materials and methods

### 2.1 Collection of cysts and J2 of SCN

The roots of diseased red kidney beans (*Phaseolus vulgaris* L.) collected from Lan County, Shanxi Province, were gently brushed to remove cysts attached to the root surface and subsequently examined under a dissecting microscope. The collected cysts were surface-disinfected with a 0.1% NaClO solution for 1 min, followed by three rinses with sterile distilled water. They were then placed in a Baermann funnel and incubated at room temperature (25°C), with J2 being collected from the bottom of the Baermann funnel every 2-3 days.

### 2.2 Preparation of *Bacillus velezensis* A-27 fermentation broth

A single colony of A-27 grown on a plate was inoculated into 30 mL of liquid LB medium and shaken for 24 hours to prepare a seed solution. This seed solution was then inoculated into LB medium at a ratio of 1:100 and shaken at 28°C and 150 rpm for 72 hours until the OD_600_ value to 1.3.

### 2.3 Plant material and treatments

The red kidney bean cultivar Pinjinyun no. 4 was chosen as the experimental material. The seeds were surface-disinfected with 0.5% NaClO for 10 min, rinsed with sterile distilled water, air-dried, and then sown directly into plastic pots filled with sterilized soil and vermiculite. Upon reaching the vegetative cotyledon (Vc) stage, the seedlings were allocated into four treatments: (1) irrigation with 5 mL of LB medium only (CK), (2) root irrigation with 5 mL of A-27 fermentation broth (A-27), (3) inoculation with 2000 SCN J2 (J2), and (4) root irrigation with A-27 fermentation broth followed by inoculation with 2000 SCN J2 after 3 days (A-27 + J2). The plants were maintained at a day/night temperature of 25/20°C, with relative humidity between 60-80%, 16 hours of daily light, and were irrigated with a 1/4 strength Hoagland nutrient solution throughout the growth cycle. Root samples from the treated red kidney bean plants were collected for proteomic and transcriptomic analysis at 3, 7, 14, 21, and 28 days after inoculation (DAI). Each collection involved sampling five plants per treatment, with each treatment replicated three times.

### 2.4 Extraction, TMT labeling, and identification of proteins

Frozen samples were transferred into low protein binding tubes (2 mL Eppendorf), and 500 µL of extraction buffer was added to each sample with steel beads. The samples were ground at 60 Hz for 2 min. Subsequently, additional extraction buffer was added to achieve a final volume of 1 mL. Tris-phenol buffer was added to the mixtures, which were then mixed for 30 min at 4°C. Subsequently, the mixtures were centrifuged at 7,100 rpm for 10 min at 4°C to collect the phenol supernatants. These supernatants were mixed with 5 times their volume of 0.1M cold ammonium acetate-methanol buffer and precipitated overnight at -40°C. After precipitation, the samples were centrifuged at 12,000 rpm for 10 min to collect the precipitate, which was washed with cold methanol and gently mixed. This washing step was repeated once. Subsequently, methanol was replaced with acetone, and the washing step was repeated twice to remove any methanol contamination. The samples were then centrifuged at 12,000 rpm for 10 min at 4°C to collect the precipitate, which was dried at room temperature for 3 min and dissolved in a lysis buffer supplemented with 1 mM PMSF for 3 min. Finally, the samples underwent an additional centrifugation step at 12,000 rpm for 10 min to ensure the complete removal of any remaining precipitate.

The protein concentration was determined using the Bradford assay (Bio-Rad) with bovine serum albumin (BSA) as the standard. The protein samples were then aliquoted and stored at -80°C for further analysis. For TMTpro labeling, the lyophilized samples were resuspended in 100 μL of 100 mM TEAB (pH 8.5) and 40 μL of each sample were transferred into a new tube for labeling. Anhydrous acetonitrile was added to the TMT reagent vial at room temperature. The reagents were centrifuged, dissolved for 5 min, mixed and centrifuged, repeating this step once. Subsequently, 10 μL of the TMTpro label reagent was added to each sample for mixing. The tubes were then incubated at room temperature for 1 hour. Finally, 5 µL of 5% hydroxylamine was added to each sample and incubated for 15 min to quench the reaction.

The labeled peptide solutions were freeze-dried and stored at -80°C. The separation process was conducted using an Agilent 1100 HPLC System equipped with an Agilent Zorbax Extend -C18 column (5 μm, 150 mm × 2.1 mm). A gradient of mobile phases A (2% acetonitrile in HPLC water) and B (90% acetonitrile in HPLC water) were used. The solvent gradient was set as follows: 0-8 min, 98% A; 8-8.01 min, 98%-95% A; 8.01-48 min, 95%-75% A; 48-60 min, 75-60% A; 60-60.01 min, 60-10% A; 60.01-70 min, 10% A; 70-70.01 min, 10-98% A; 70.01-75 min, 98% A. Tryptic peptides were separated at a flow rate of 300 μL/min and monitored at 210 nm. Samples were collected for 8-60 min, with the eluent collected in centrifugal tubes 1-15 every minute in sequence. The peptides were recycled in this order until the end of the gradient. Subsequently, the separated peptides were freeze-dried for mass spectrometry analysis.

### 2.5 LC-MS/MS analysis and protein annotation

Changes in the proteome of red kidney bean roots from four different treatments were analyzed using TMT-LC/MS-MS technology. All analyses were conducted using a Q-Exactive HF mass spectrometer (Thermo, USA) equipped with a Nanospray Flex source (Thermo, USA). Samples were loaded and separated on a C18 column (25 cm × 75 µm) using an EASY-nLCTM 1200 system (Thermo, USA). The flow rate was 300nL/min with a linear gradient over 240 min (0-160 min, 3%-21% B; 160-220 min, 21%-42% B; 220-230 min, 42%-90% B; 230-240 min, 90% B; where mobile phase A= 0.1% FA in water and B = 0.1% FA in 80% ACN and 19.9% water). Full MS scans were conducted in the mass range of 300-1650 m/z with a resolution of 120000 and an AGC target value of 3e6. The 15 most intense peaks in the MS were fragmented using higher-energy collisional dissociation (HCD) with a collision energy of 30 MS/MS spectra were obtained with a resolution of 30000, an AGC target of 1e5, and a maximum injection time of 80 ms. The Q Exactive HF dynamic exclusion was set to 30.0 s and the analysis was run in positive mode.

The LC-MS/MS raw data were analyzed in MaxQuant (Version 1.6.17.0) with a global false discovery rate (FDR) of 0.01 and a minimum of 3 peptides for protein group quantification. In order to be identified as substantially differentially expressed, searches were based on quantitative data from the UniProt library with fold change of ≥1.5 or ≤1/1.5, expression levels at p<0.05, and a false discovery rate of less than 1%. Gene Ontology (GO) annotations for the *Phaseolus vulgaris* proteome were obtained from the UniProtKB at the Gene Ontology Annotation (GOA) database. Identified protein IDs were converted to UniProt IDs and then annotated to GO IDs. Proteins without UniProt GOA annotations underwent further GO function analysis using the InterPro database through protein sequence alignment. Metabolic pathways were annotated using the Kyoto Encyclopedia of Genes and Genomes (KEGG) database and mapped to the KEGG pathway database using the KEGG mapper online service tool.

### 2.6 RNA extraction and transcriptional analysis

Total RNA was extracted from red kidney bean roots subjected to four treatments at 3, 7, 14, 21, and 28 DAI with SCN J2. The RNA extraction was performed using the RNA pure Plant Kit (Tiangen, Beijing, China) following the manufacturer’s instructions. Transcriptional analysis of differentially expressed genes (DEGs) encoding proteins involved in α-LeA metabolism was conducted using qRT-PCR. Gene-specific primers were designed using SnapGene 4.0 (**Table 1**). The housekeeping gene *Actin* (GenBank accession: KF033476) from red kidney beans was served as the internal control. The qRT-PCR was carried out with a 20 µL reaction system, including 2 µL cDNA template (100 ng), 0.5 µL (10 mM) forward and reverse primers each, 12 µL 2 × SYBR Premix ExTaq (TaKaRa, Beijing, China), and 5 µL double-distilled H_2_O. Cycling conditions for the qRT-PCR reactions were as follows: 95°C for 30 s, followed by 30 cycles of 95°C for 5 s and 60°C for 20 s, 95°C for 60 s, 55°C for 30 s, and 95°C for 30 s. The qRT-PCR data were analyzed using the 2 ^-ΔΔCt^ relative quantification method. The significant difference between treatments was determined using the Statistical Investigation Data Processing System. Each experiment was conducted with three biological replicates.

**Table 1:** Primers and their sequences used in the qRT-PCR analysis.

Gene	Primer	Sequence (5’-3’)
*ACX*	*ACX* F	GTGGCGGCGGAGCAGTTG
	*ACX* R	AGGTGCGGATTGGCGTTGAAG
*AOC*	*AOC* F	GCTGTTACAGGAGGCTCTGGAATC
	*AOC* R	AGGTTCAACAGGCTTCCCAAGTAG
*PLA1*	*PLA1* F	TGGTGAACGCCGAAGGAGGAG
	*PLA1* R	ATCCGCAGCATCTCGTACACAAC
*AOS*	*AOS* F	CCGACGAGGTGAGGGCGATC
	*AOS* R	GAACTCCATGTCAGCGAGCAGAAG

Letters FandRindicate the forward and reverse primers.

### 2.7 Measurement of JA contents in red kidney beans roots

Total JA content was extracted from 0.5 g fresh root samples collected under four different treatments using the ELISA method. The roots were immediately frozen in liquid nitrogen, finely ground into a homogenate, and then mixed with 200 μL of PBS (pH 7.4). Following this, the mixture was then centrifuged for 20 min at 2000 rpm, and the supernatant was carefully collected for further analysis. For testing, 10 μL of the sample was mixed with 40 μL of sample diluent in a testing sample well. Subsequently, 100 μL HRP-conjugate reagent was added to each well, covered with an adhesive strip, and incubated for 60 min at 37°C. After incubation, each well was aspirated and washed four times with wash solution (400 μL per wash). Following the washes, chromogen solution A (50 μL) and chromogen solution B (50 μL) were added to each well, gently mixed, and incubated for 15 min at 37°C. Stop solution (50 μL) was then added to each well, resulting in a color change from blue to yellow. The plate was gently tapped to ensure thorough mixing. Optical density (O.D.) was measured at 450 nm using a microtiter plate reader within 15 min. A concentration-OD plot was created in an Excel worksheet, with the concentration of the standard solution on the x-axis and the corresponding OD values on the y-axis. A linear regression curve was calculated for the standard solution, and the concentration values of each sample were determined based on the curve equation.

### 2.8 Statistical analysis

Statistical analysis was conducted using a two-way ANOVA in GraphPad Prism9. Significant differences between group means were determined using the Tukey’s multiple comparisons test at a significance level of *p*<0.05 (Wang et al., 2020).

## 3 Results

### 3.1 Protein identification by TMT analysis

Data from three biological replicates were examined using TMT-LC/MS-MS technology, and proteins were identified by querying a *Phaseolus vulgaris* protein database. A total of 87,785 effective spectra (out of 350,121) were detected across the four groups, leading to the identification 8,186 distinct peptides (out of 50,022) through proteomic analysis.

**Figure 1:**
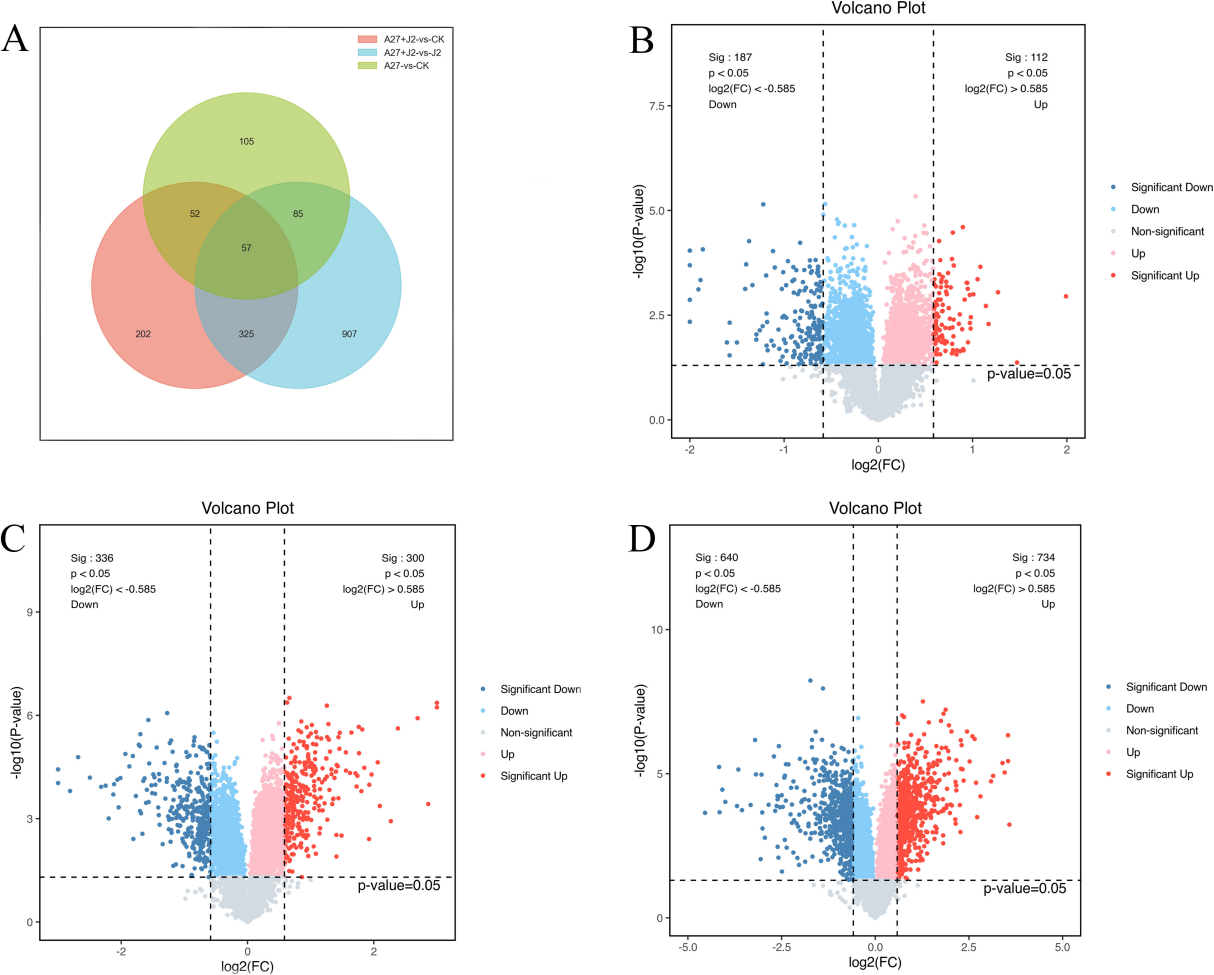
Venn diagram of DEPs responsive in A-27 *vs* CK, (A-27 + J2) *vs* CK and (A-27 + J2) *vs* J2 **(A)**; Volcano plot shows DEPs that up and down regulated in A-27 *vs* CK **(B)**, (A-27 + J2) *vs* CK **(C)**, and (A-27+J2) *vs* J2 **(D)**.

DEPs were selected based on a threshold of more than 1.5-fold change and p<0.05. Following these criteria, 299 DEPs were identified in red kidney bean roots, with 112 (49.2%) showing increased abundance and 187 (50.8%) showing decreased abundance between A-27 *vs* CK treatment. Additionally, 1,374 DEPs were identified in red kidney bean roots between A-27 + J2 *vs* J2 treatment, with 734 (47.6%) showing increased abundance and 640 (52.4%) showing decreased abundance. In the case of A-27 + J2 *vs* CK treatment, 636 DEPs were identified, with 300 up-regulated and 336 down-regulated proteins (**Figure 1**).

### 3.2 GO and KEGG annotations of DEPs

**Figure 2:**
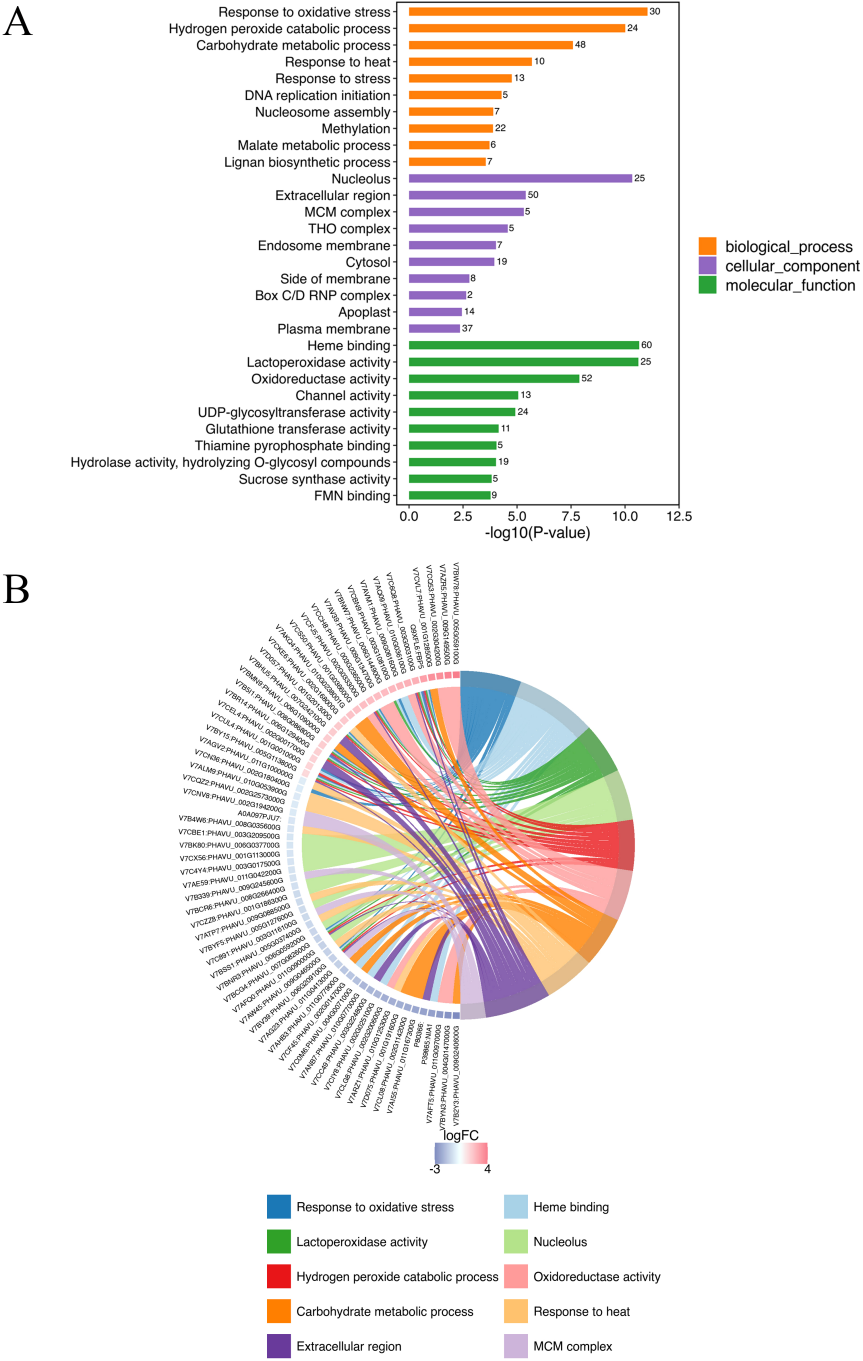
Top GO annotations of DEPs in (A-27 + J2) *vs* J2 **(A)** and GO Chord of top 10 GO terms **(B)**.

GO annotation was conducted all DEPs in A-27 + J2 *vs* J2, resulting in a total of 856 annotated GO terms. These terms encompassed 359 biological processes, 88 molecular functions, and 409 cellular components. Among the top 10 terms identified across the three categories, notable biological processes included response to oxidative stress (30 DEPs), hydrogen peroxide catabolic process (24 DEPs), carbohydrate metabolic process (48 DEPs), and response to heat (10 DEPs). The cellular components with the highest number of annotated proteins were nucleolus (25 DEPs), extracellular region (50 DEPs), and MCM complex (5 DEPs), while the molecular functions enriched with the most proteins were heme binding (60 DEPs), lactoperoxidase activity (25 DEPs), and oxidoreductase activity (52 DEPs) (**Figure 2**; **Supplementary Table S1**).

**Figure 3:**
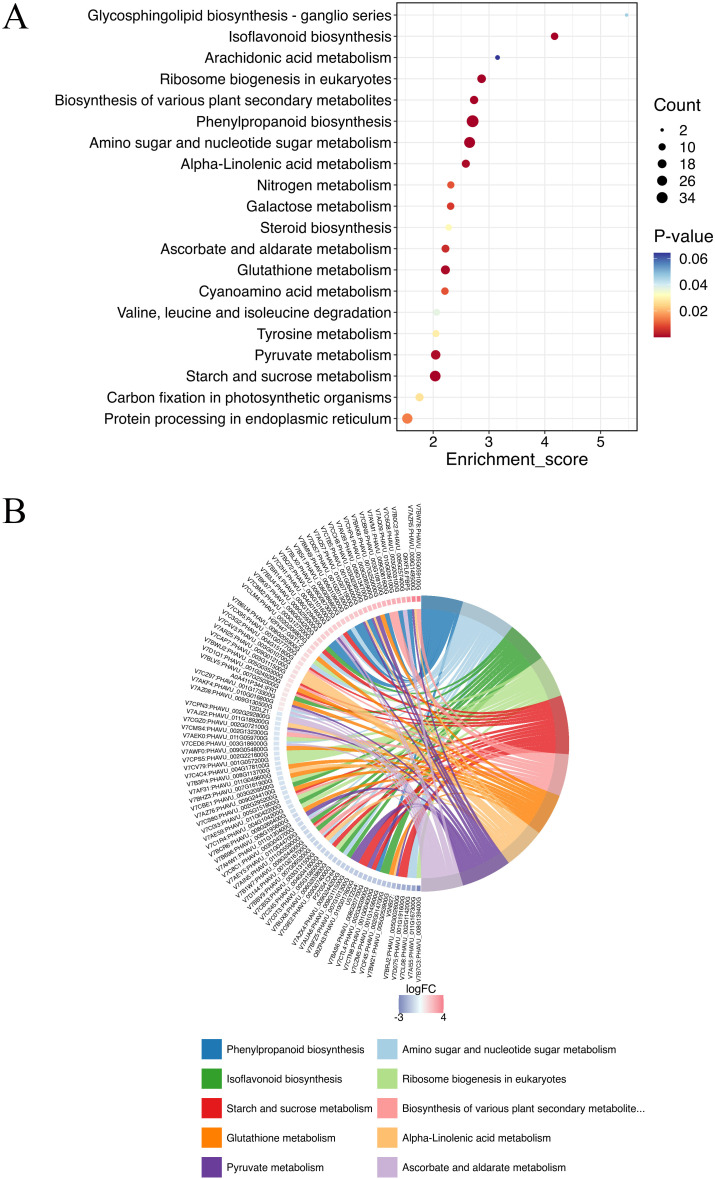
Top KEGG enrichment analysis of all DEPs in (A-27 + J2) *vs* J2 **(A)**, and KEGG Chord of top 10 KEGG terms **(B)**.

The KEGG analysis identified 118 pathways, focusing on the synthesis and metabolism of various biological substances. Some of these metabolic pathways may be related to plant disease resistance, such as isoflavonoid biosynthesis (pvu00943), arachidonic acid metabolism (pvu00590), phenylpropanoid biosynthesis (pvu00940), glutathione metabolism (pvu00966), pyruvate metabolism (pvu00640), biosynthesis of various plant secondary metabolites (pvu00999), and α-LeA metabolism (pvu00592) (**Figure 3**; **Supplementary Table S2**). Further investigations are needed to explore additional metabolic pathways associated with other DEPs.

### 3.3 Validation of the DEPs in α-LeA metabolism pathway by qRT-PCR at transcriptional level

The study revealed that *Bacillus velezensis* A-27 increased red kidney bean resistance to SCN by enhancing 14 DEPs involved in the α-LeA metabolism pathways. To confirm these results, 4 key genes including PHAVU_008G052100g (*PLA1*), PHAVU_003G111500g (*AOC*), PHAVU_002G254200g (*ACX*), and PHAVU_003G010700g (*AOS*) (**Supplementary Table S3**), which are involved in the α-LeA metabolism pathway, were selected for qRT-PCR analysis.

**Figure 4:**
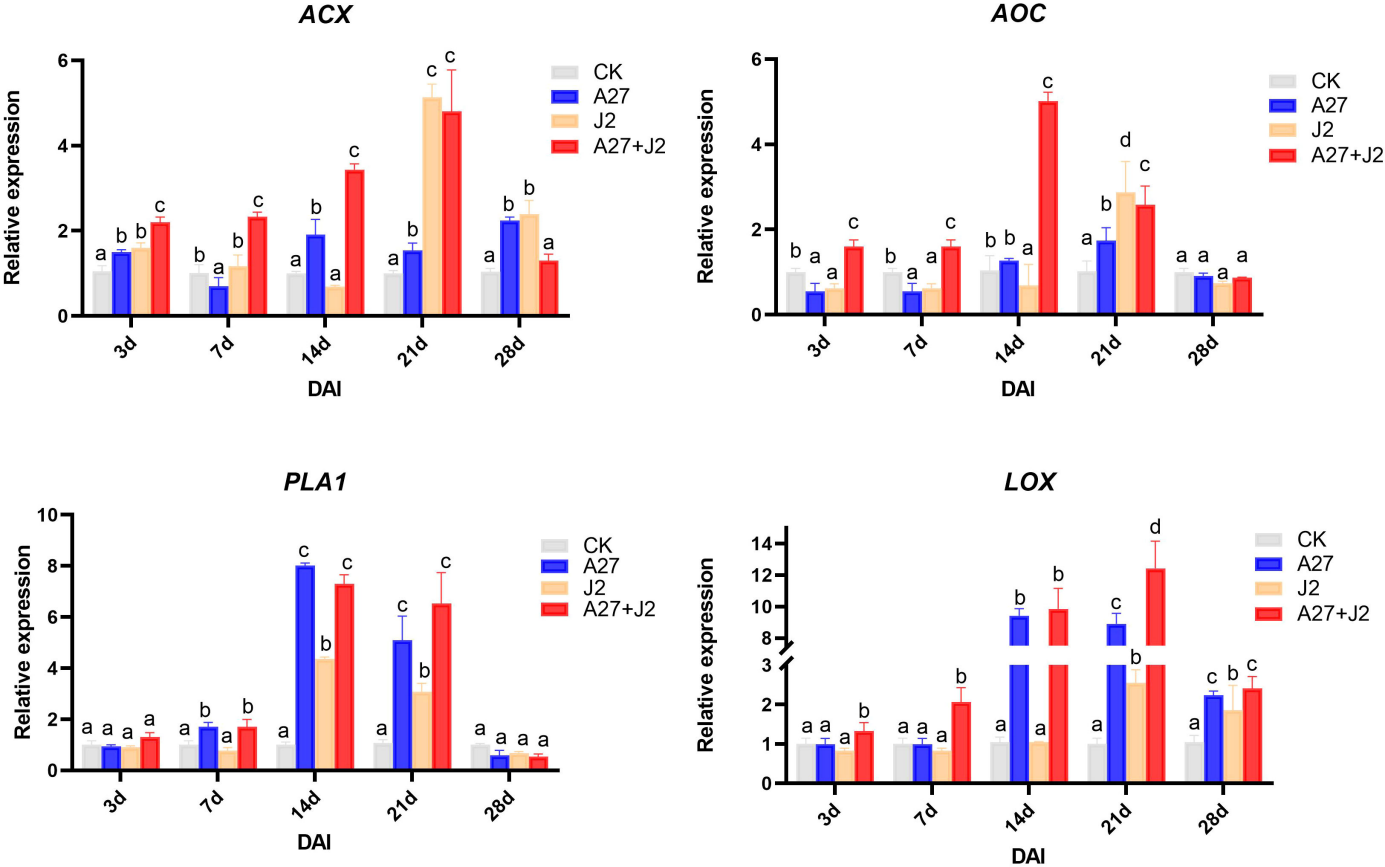
Quantitative RT-PCR analysis of 4 key genes involved in the α-LeA metabolism pathway in the root. Gene expression levels were analyzed from four treatments: CK, A-27, J2, and A-27 + J2 at 3, 7, 14, 21, and 28 DAI. Data are represented as mean ± standard deviation (SD) of three biological replicates. Different letters a, b, c, and d indicate the significant difference set at p value < 0.05.

At 3 DAI, there was no significant change in the *PLA1* expression level across treatments. However, by 7 DAI, when J2 feeding sites began to form, *PLA1* expression significantly increased in the A-27 and A-27 + J2 treatments. By 14 DAI, *PLA1* expression was significantly upregulated in all treatments, with the highest level observed at 7.9-fold higher. Expression levels gradually returned to normal by 21 DAI, with no significant differences by 28 DAI. The *AOC* gene expression showed significant differences among treatments at 3 DAI, with significant downregulation in the A-27 and J2 treatments, while significant upregulation was observed in the A-27 + J2 treatment. This trend continued at 7 DAI, with *AOC* expression in the A-27 + J2 treatment being 4.5-fold higher than the control by 14 DAI. The *HPL* gene was significantly upregulated in the A-27 + J2 treatment at 3 DAI, with expression sharply increasing in the A-27 and A-27 + J2 treatments by 14 DAI, reaching 9.7-fold higher and 9.9-fold higher, respectively. The A-27 + J2 treatment peaked at 12.3-fold higher by 21 DAI. In contrast, the *ACX* expression downstream of the JA synthesis pathway varied significantly across stages. In the A-27 + J2 treatment, *ACX* was significantly upregulated at 3, 7, 14, and 21 DAI, reaching its highest level of 4.9-fold higher at 21 DAI. Similarly, the J2 treatment group showed a peak in *ACX* expression at 5.2-fold higher at 21 DAI (**Figure 4**).

### 3.4 Measurement of JA contents in red kindy bean roots

**Figure 5:**
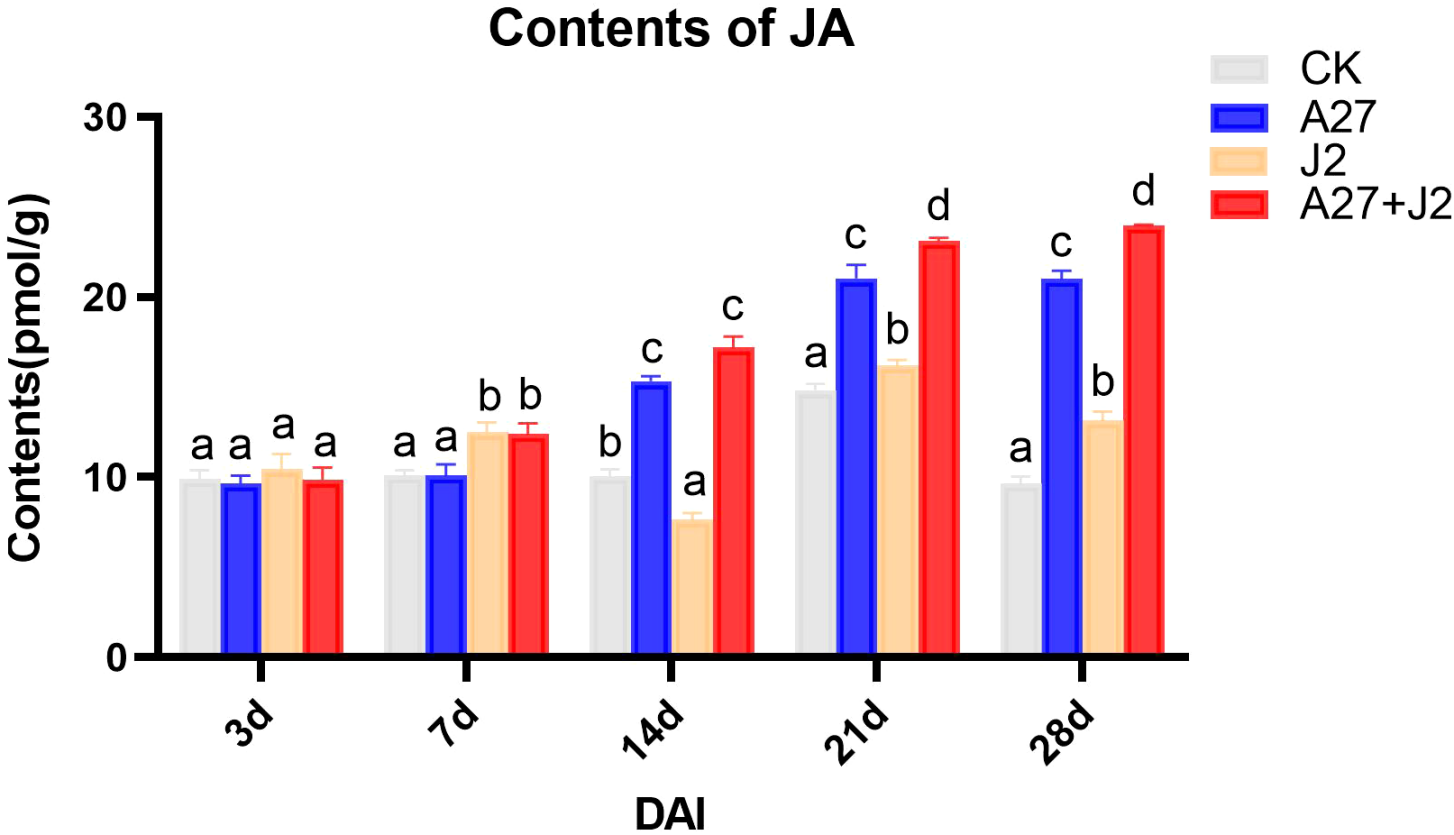
Contents of JA in red kindy beans roots at different times intervals. Data are represented as mean ± standard deviation (SD) of three biological replicates. Different letters a, b, c, and d indicate the significant difference set at p value < 0.05.

The study investigated the impacts of genetic modifications on genes related to the α-LeA metabolism pathway, specifically examining whether these modifications would result in a substantial change in JA concentrations in red kidney bean roots under four different treatments. JA levels were measured using ELISA, with the results are shown in **Figure 5**.

Overall, the JA content in red kidney bean roots exhibited a gradual increase over time across all three treatment groups, with significant differences observed from 14 DAI. No significant changes in JA content were detected at 3 DAI and 7 DAI across different treatments. However, by 14 DAI, both the A-27 and A-27 + J2 treatment exhibited a significant increase in JA content, which was consistently maintained until 28 DAI. Furthermore, starting from 21 DAI, the J2 treatment group also demonstrated a significant increase in JA content.

## 4 Discussion

The severity of SCN disease has progressively worsened due to continuous cultivation in red kidney bean planting areas of Shanxi Province, China. Previous research conducted in our laboratory has classified the SCN population infecting red kidney beans as race 5 (Riggs and Schmitt, 1988). Our results demonstrated that the cultivar Pinjinyun no. 4, when treated with A-27 in combination with SCN J2, exhibited increased fresh plant weight and root weight, along with a reduced number of cysts in the roots compared to CK (**Supplementary Figure S1**). In this study, we aim to investigate the mechanism by which the PGPR *B. velezensis* A-27 enhances the growth and resistance of red kidney beans to SCN. This research seeks to provide a foundation for the application of *B. velezensis* A-27 in the prevention and management of SCN infestations in various leguminous crops.

TMT technology was used to compare the total proteins in red kidney beans root systems to investigate the induction of resistance to SCN by A-27 in this study. SCN J2 usually takes 3-7 days to locate host root by recognizing root exudate signals and establishing feeding sites (Duan et al., 2011). Consequently, root samples were collected at 7 DAI for TMT proteomics analysis. The results revealed 1,374 DEPs in the A-27 + J2 *vs* J2 treatment, which is significantly higher than the 299 DEPs observed in the A-27 *vs* CK treatment and the 636 DEPs found in the A-27 + J2 *vs* CK treatment. This suggests that the combined infection of A-27 and J2 on plants enhances the complexity of the plant immune response, adding significant value to our research. Interestingly, despite both A-27 + J2 *vs* J2 and A-27 *vs* CK treatments involving A-27 treatment on plant roots, KEGG analysis highlighted the α-LeA metabolism pathway as a key pathway. However, there were 14 DEPs associated with the former and only 3 with the latter, indicating that J2 infection complicates JA biosynthesis in plants.

**Figure 6:**
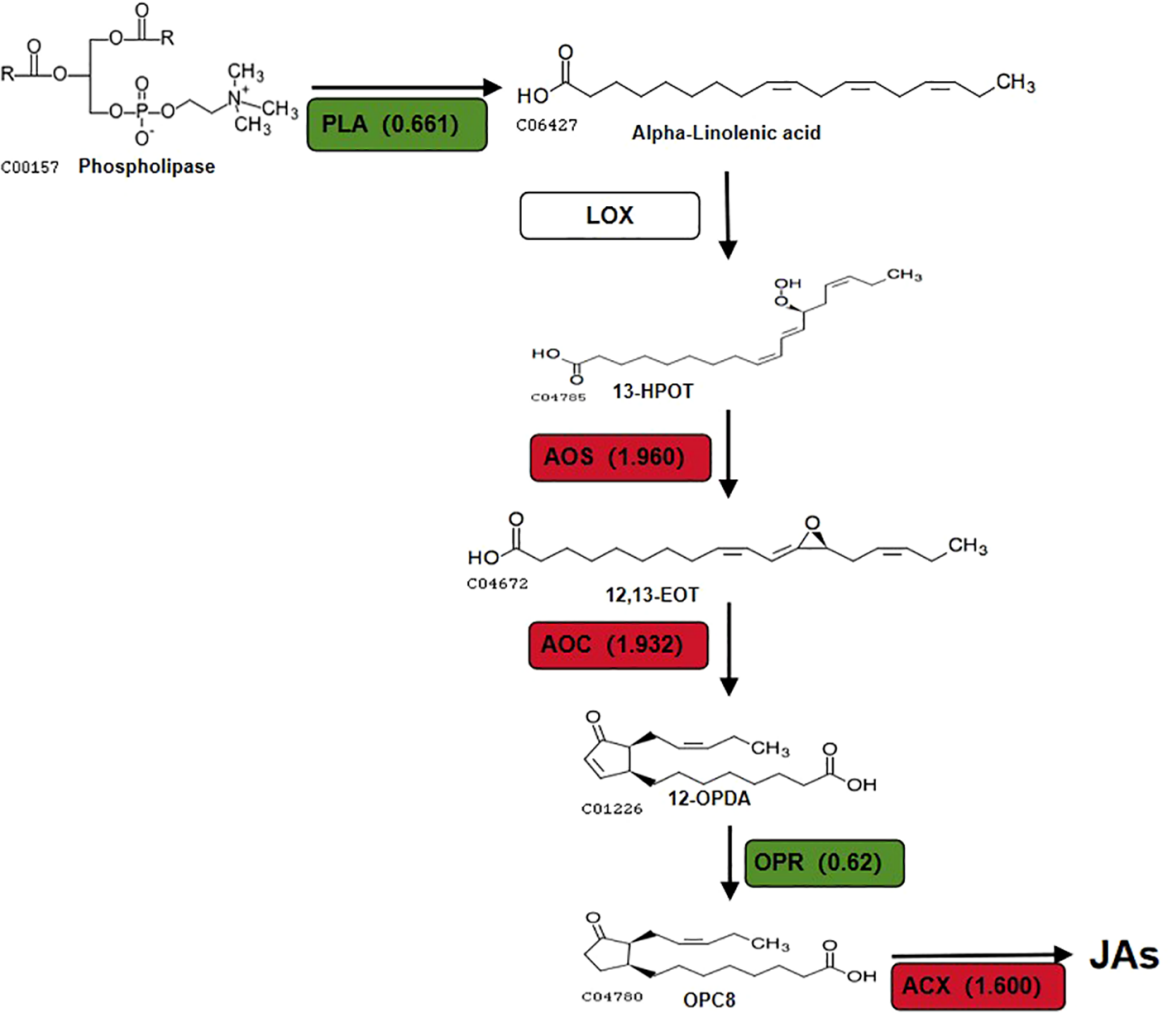
DEPs that involved in JAs biosynthesis pathway in (A-27 + J2) vs J2. Different colors and numbers show the fold-changes in protein abundance (Han et al., 2016) based on the TMT data.

The focus of this study will be to discuss key DEPs related to the α-LeA biosynthesis pathway. The synthesis of JAs through the α-LeA pathway initiates with the conversion of phosphatidylcholine from chloroplasts or plastid membranes via phospholipases (PLAs) (Abdelkareem et al., 2017). Notably, a *PLA* gene in the red kidney bean roots exhibited significant expression changes, consistent with alterations at the transcriptome level, potentially offering new insights into the role of A-27 as a PGPR that enhances plant resistance by stimulating JA biosynthesis. The LOX pathway, a crucial secondary metabolic pathway in plants, involves the peroxidation of polyunsaturated fatty acids released from glycerides to produce peroxide fatty acids. One pathway involves the oxidation of 13-HPOT by HPL, resulting in the production of short-chain volatile aldehydes, alcohols, or oxygenated acids as defense responses to biotic (bacteria, fungi, or viruses) or abiotic (mechanical damage, high temperature, drought stresses) stressors (Heitz et al., 2019). Another pathway includes the conversion of 13-HPOT to 12-oxo-phytodienoic acid (12-OPDA) by AOS and AOC, followed by the reduction of 12-OPDA to JA and its derivatives by OPR and ACX in the peroxisomes (Browse, 2009; Gfeller et al., 2010; Lahari et al., 2024). Although no significant changes were observed in LOX in the A-27 + J2 *vs* J2 treatment, significant changes were detected in 2 genes encoding AOS, 2 genes encoding AOC, and 1 gene encoding HPL (**Figure 6**).

This results in an increase in volatile aldehydes and alcohols produced by HPL catalysis without altering the concentration of 13-HPOT, potentially repelling SCN from plant roots and reducing their harmful impact on plants. This finding offers a novel perspective on the enhanced resistance of A-27 to SCN. Furthermore, the production of 12-OPDA, catalyzed by AOS and AOC, has been associated with determining plant susceptibility to PPN (Carty et al., 2023).

Four key genes in the α-LeA biosynthesis pathway (*PAL1, AOS, AOC, ACX*) were regulated at various stages after treatment, leading to the biosynthesis and accumulation of JA in red kidney bean roots. The induction process and abundance of JA varied among the four treatments. Both SCN and *B. velezensis* A-27, whihc are known as parasites of soybean, can induce the plant’s resistance response through pattern-triggered immunity (PTI), leading to the production of non-specific defense responses in plants (Sathiyamoorthy et al., 2010). Among the four treatments, we investigated the accumulation of JA and found a more advanced accumulation process in red kidney beans treated with A-27 compared to untreated ones. This indicates significant differences in the processes of inoculating A-27 and J2 infection in stimulating plant resistance. Upon successful colonization of red kidney bean roots by A-27, a rapid plant immune response is activated, enhancing the transcription of AOS and AOC, and promoting JA biosynthesis compared to the control and J2 treatments. Interestingly, at 7 DAI, JA accumulation was initially observed in both J2 and A-27 + J2 treatments; however, by 14 DAI, as J2 established feeding sites and successfully invaded, JA content decreased significantly. This decrease may be attributed to effectors released during the successful J2 infection, which possess mechanisms to suppress plant defense responses. In contrast, samples treated with A-27 exhibited a continuous increase in JA content. Previous studies have suggested that, in addition to activating plant defense responses, JA may influence the recognition and infection behavior of nematodes on plants, as well as their developmental processes (Schouteden et al., 2017), potentially hindering continuous J2 infection.

This study presents a novel discovery showing that *B. velezensis* A-27 induces the biosynthesis of JA in red kidney beans, resulting in the activation of ISR. In addition to the significant increase in JA levels, the stimulation of intermediate metabolites, including volatile aldehydes and 12-OPDA along the metabolic pathway, may also contribute to enhancing the plants’ defense against nematode infections. Analysis of key enzymes and transcriptomes in the α-LeA metabolic pathway revealed that *B. velezensis* A-27 enhances red kidney bean resistance to SCN by promoting JA biosynthesis. However, further research is needed to fully elucidate the intricate mechanism by which *B. velezensis* A-27 induces systemic acquired resistance (SAR) in leguminous crops, like red kidney beans to improve their resistance against SCN. Overall, *B. velezensis* A-27 demonstrates potential as a biocontrol agent (BCA) for SCN management.

## 5 Conclusions

This study investigated the molecular mechanisms by which the PGPR *Bacillus velezensis* A-27 enhances the resistance of red kidney beans to SCN by modulating the JA biosynthesis pathway. Through the use of TMT proteomics technology, a total of 1,374 DEPs were identified in the roots of red kidney beans treated with A-27 + J2 compared to those treated with J2 alone. These results highlighted the significant role of the JA biosynthesis pathway in enhancing disease resistance. Validation through qRT-PCR confirmed the upregulation of 4 key genes in the JA biosynthesis pathway, and ELISA demonstrated a significant increase in JA content in the roots. This study reveals that *B. velezensis* A-27 induces ISR in red kidney beans by modulating the JA biosynthesis pathway, thereby enhancing the plant’s defense against SCN.

## Data Availability

The original contributions presented in the study are publicly available. This data can be found here: NGDC, accession number PRJCA030088, https://ngdc.cncb.ac.cn/omix/preview/oecEpt3Q.
