# Supplementary figures and images for: Research on the mechanism of Bacillus velezensis A-27 in enhancing the resistance of red kidney beans to soybean cyst nematode based on TMT proteomics analysis
Source: Front Plant Sci. 2024 Sep 23;15:1458330. doi: 10.3389/fpls.2024.1458330 (PMC11456435; doi:10.3389/fpls.2024.1458330)

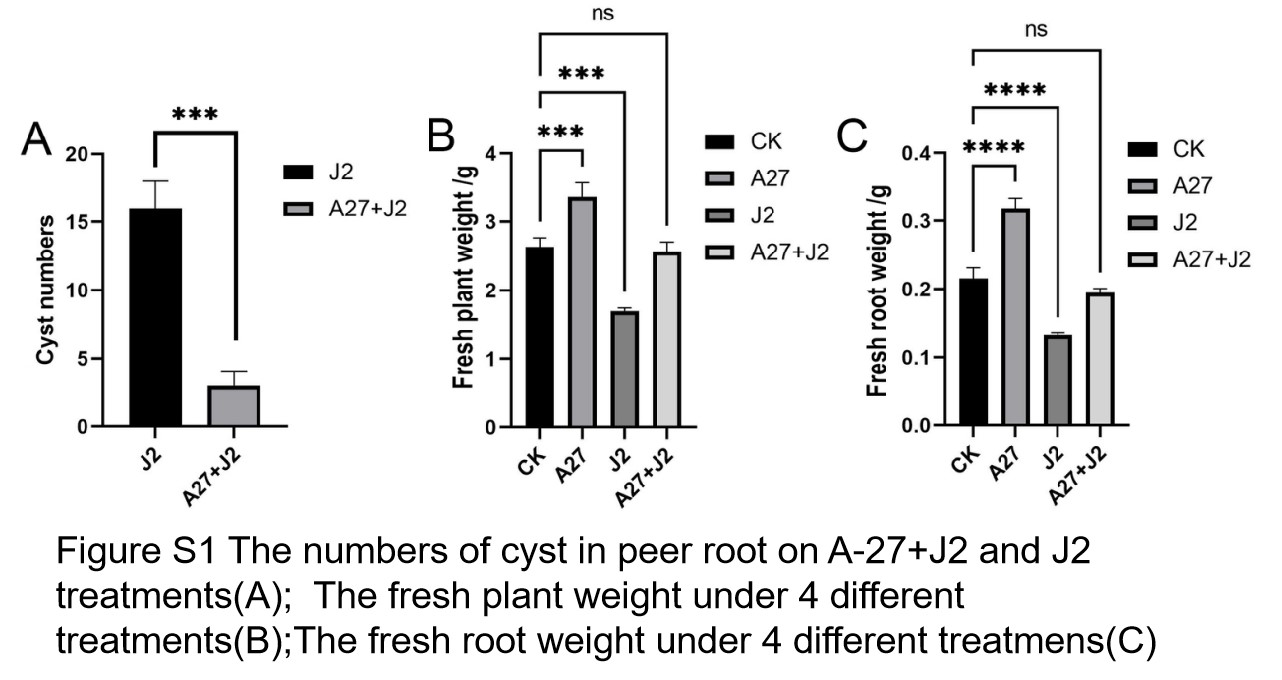

Supplement: Supplementary file 1 [file Image1.jpeg]
